# Long‐term response to osimertinib in elderly patients with lung adenocarcinoma harbouring de novo *EGFR* T790M: a case report and literature review

**DOI:** 10.1002/rcr2.759

**Published:** 2021-05-04

**Authors:** Toshiyuki Sumi, Koki Kamada, Naoki Shijubou, Yuichi Yamada, Hisashi Nakata, Yuji Mori, Hirofumi Chiba

**Affiliations:** ^1^ Department of Pulmonary Medicine Hakodate Goryoukaku Hospital Hakodate Japan; ^2^ Department of Respiratory Medicine and Allergology Sapporo Medical University School of Medicine Sapporo Japan

**Keywords:** De novo T790M, epidermal growth factor receptor, lung adenocarcinoma, osimertinib

## Abstract

Osimertinib is a potent and irreversible epidermal growth factor receptor (EGFR) tyrosine kinase inhibitor (TKI) that selectively acts on both EGFR‐sensitive and *EGFR* T790M‐resistant mutations. Patients with pre‐treatment *EGFR* T790M mutations (de novo *EGFR* T790M) respond poorly to existing EGFR‐TKIs, whereas osimertinib has positive effects. However, the safety data for first‐line osimertinib treatment in patients aged >75 years are insufficient. We treated two elderly patients with de novo *EGFR* T790M mutations with osimertinib as the first‐line therapy. We found that the first‐line treatment with osimertinib was safe and resulted in a long‐term response in elderly patients with de novo *EGFR* T790M‐mutated lung adenocarcinoma.

## Graphical abstract

There are no safety data on the first‐line osimertinib treatment in patients aged 75 years or older, defined as the elderly in Japan, and very few reports exist on the efficacy of first‐line therapy for de novo epidermal growth factor receptor (EGFR) T790M‐positive lung cancer in elderly patients. We report a safe and long‐term response to the first‐line treatment with osimertinib in de novo EGFR T790M‐positive patients older than 75 years.

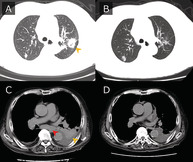

## Introduction

Osimertinib is a potent irreversible epidermal growth factor receptor (EGFR) tyrosine kinase inhibitor (TKI) that selectively acts on both *EGFR*‐sensitive and *EGFR* T790M‐resistant mutations. Osimertinib is currently available for the first‐line treatment of *EGFR* mutation‐positive lung cancer and second‐line treatment for lung cancer harbouring an *EGFR* T790M mutation; however, a percentage of de novo *EGFR* T790M mutations exist before starting treatment [1]. Although the first‐ and second‐generation TKIs for de novo *EGFR* T790M‐positive lung adenocarcinoma are ineffective, osimertinib is known to be effective [2, 3, 4, 5]. However, there are no safety data on the first‐line osimertinib treatment in patients aged 75 years or older, defined as the elderly in Japan, and very few reports exist on the efficacy of first‐line therapy for de novo *EGFR* T790M‐positive lung cancer in elderly patients [2, 3]. We report a safe and long‐term response to the first‐line treatment with osimertinib in de novo *EGFR* T790M‐positive patients older than 75 years.

## Case Report

**Figure 1:**
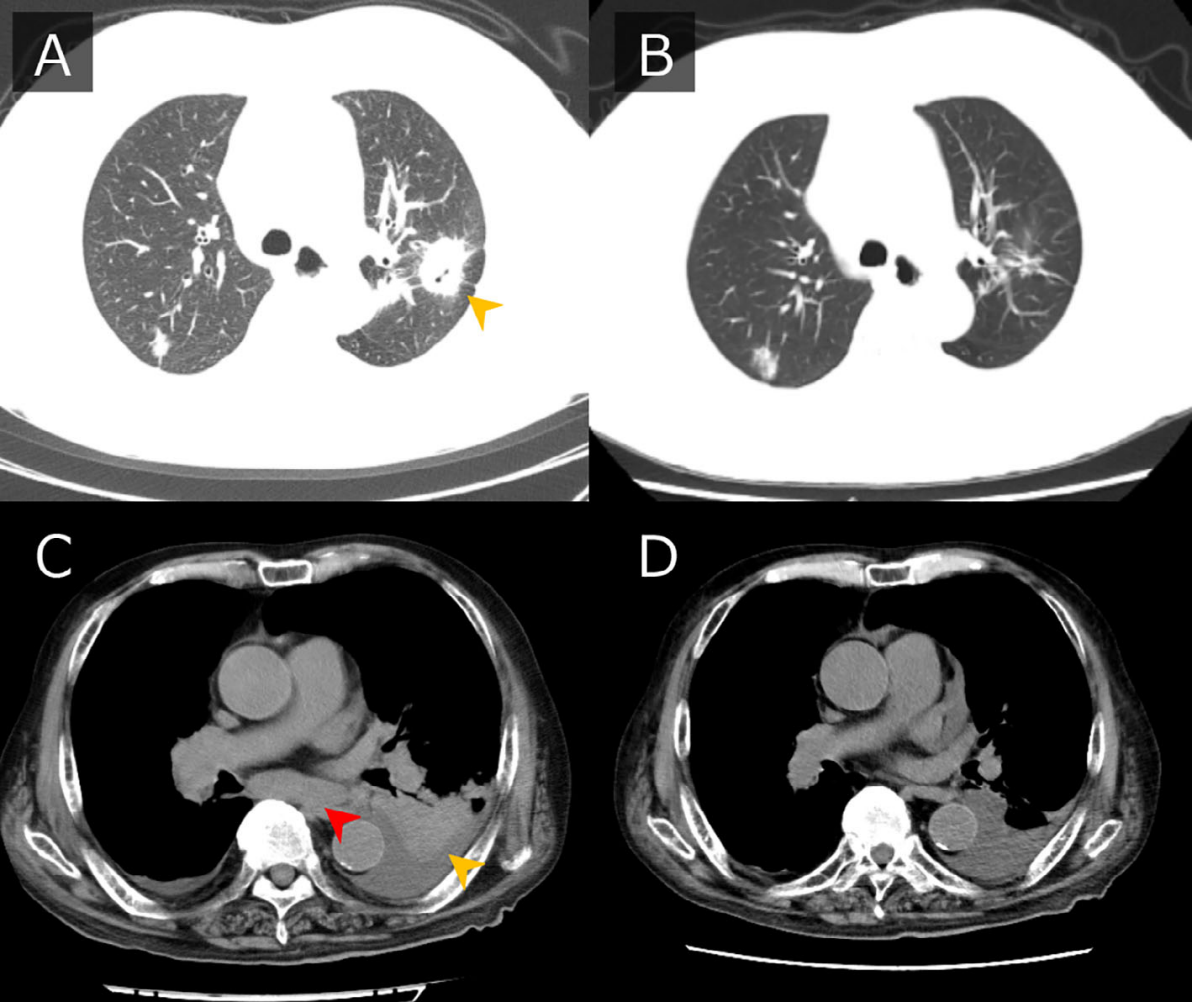
Computed tomography (CT) findings before the start of osimertinib treatment and at the time of the best response. In case 1, chest CT showed a mass (indicated by the yellow arrowhead) in the left upper lobe (A) that almost disappeared after three months of treatment (B). In case 2, chest CT showed a mass (indicated by the yellow arrowhead) in the left lower lobe and sub‐carinal lymph node metastasis (indicated by the red arrowhead) (C). After two months of treatment, the mass shrank and lymphadenopathy disappeared (D).

Case 1 was of a 76‐year‐old woman with hypertension, pre‐existing osteoarthritis of the knee, and performance status 1 on initial examination. Chest computed tomography revealed a mass in the left upper lobe and multiple pulmonary metastases in both lungs. The primary tumour was biopsied and diagnosed as adenocarcinoma cT4N3M1c stage 4B (metastases in the brain, lungs, bones, and adrenal glands) with *EGFR* exon 21 L858R and exon 20 T790M. Osimertinib at 80 mg/day was started, but the dose was reduced to 40 mg/day two weeks later because of a grade 2 skin rash. Subsequently, the tumour shrank and the best therapeutic effect was a partial response (Fig. 1A, B). The patient has been responding to osimertinib for 26 months to date.

Case 2 was of an 89‐year‐old man with a history of hypertension, chronic heart failure, and lumbar spinal canal stenosis; he was found to have performance status 2 on initial examination. Chest computed tomography showed a mass and pleural effusion in the left lower lobe. Biopsy of mediastinal lymph nodes revealed adenocarcinoma cT2bN3M1c stage 4B (metastases in the pleura, bone, and liver) with *EGFR* exon 19 deletion and exon 20 T790M. Osimertinib at 80 mg/day was started. There were no significant side effects, the tumour shrank, the performance status improved to 1, and the best response was a partial response (Fig. 1C, D). Osimertinib has been effective for 24 months to date.

## Discussion

We found that the first‐line treatment with osimertinib was safe and resulted in a long‐term response in elderly patients with de novo *EGFR* T790M‐mutated lung adenocarcinoma. It remains controversial whether *EGFR* T790M mutations occur de novo before treatment or if they are acquired after EGFR‐TKI treatment. In conventional DNA sequencing, the frequency of de novo *EGFR* T790M was found to be approximately 1% among EGFR‐TKI‐untreated *EGFR* mutation‐positive non‐small cell lung carcinoma [6]. A recent meta‐analysis of studies using various methods with high analytical sensitivities of 0.01–0.1% detected de novo *EGFR* T790M in 34.8–80% of patients [1]. The progression‐free survival of patients with de novo *EGFR* T790M is significantly shorter than that of patients without de novo *EGFR* T790M and has been reported to be associated with early resistance [1]. However, these studies were conducted before osimertinib was available as a first‐line treatment; thus, the results should be interpreted with caution.

As mentioned earlier, the frequency of de novo *EGFR* T790M varies depending on the test method used; however, only a few studies exist on the efficacy of osimertinib for de novo *EGFR T790M* identified before treatment. Table 1 presents details of our case along with those of previously reported cases. In the exploratory (first‐line) cohort of the AURA study that included seven patients with de novo *EGFR* T790M, treatment with osimertinib resulted in responses in six of seven patients [duration of response (DOR) 6.9–27.7 months]. Osimertinib has also been reported to affect central nervous system lesions. Noguchi et al. reported the antitumour effects of osimertinib on post‐operative brain metastasis recurrence of de novo *EGFR* T790M + exon 21 L858R disease [5]. Senoo et al. reported the antitumour effect of osimertinib against cancer meningitis with de novo *EGFR* T790M [4]. Positive results were reported following osimertinib treatment for de novo *EGFR* T790M, and this treatment is expected to have a sustained antitumour effect equivalent or superior to that against acquired *EGFR* T790M regardless of age; however, more positive case results are required to support this prediction.

**Table 1:** Characteristics and clinical outcome in patients with *de novo* T790M positive non‐small cell lung cancer.

Case	First line treatement	Dose	Sensitive mt	Positive site for T790M	Gender, M/F	Race	Age, years	Best objective response	Duration of response, months	Reference
1	Osimertinib	80 mg	L858R	Tissue	F	Asian	50	Partial response	12.2	2
2	Osimertinib	160 mg	L858R	Tissue	M	Asian	59	Partial response	27.7*	2
3	Osimertinib	80 mg	L858R	Tissue	F	Asian	60	Partial response	12.5	2
4	Osimertinib	80 mg	L858R	Tissue	F	Caucasian	49	Partial response	23.5	2
5	Osimertinib	80 mg	L858R	Tissue	F	Caucasian	61	Partial response	24.7	2
6	Osimertinib	80 mg	L858R	Plasma	F	Caucasian	77	Partial response	6.9	2
7	Osimertinib	80 mg	L858R	Plasma	F	Caucasian	65	Progressive disease		2
8	Gefitinib	80 mg	L858R	Effusion	F	Asian	78	Partial response	15*	3
9	Chemotherapy	80mg	none	Tissue, CSF	F	Asian	74	ND (Patient condition improved)	12*	4
10	Osimertinib	ND	L858R	Tissue	F	Asian	69	Partial response	4*	5
11	Osimertinib	80 mg	L858R	Tissue	F	Asian	76	Partial response	26*	Present case
12	Osimertinib	80 mg	Del 19	Tissue	M	Asian	89	Partial response	20*	Present case

* Ongoing treatment.

mt, mutation; CSF, cerebrospinal fluid; ND, not described.

As shown in the Japanese subset analysis of AURA3 and FLAURA, adverse events, including gastrointestinal symptoms, tended to be more frequent in patients treated with first‐line osimertinib than in patients previously treated with TKIs (previously treated vs. first‐line diarrhoea 34.1% vs. 56.9%; decreased appetite, 4.9% vs. 23.1%; stomatitis, 17.1% vs. 50.8%, respectively) [7, 8]. In Japanese patients aged 75 years and older treated with osimertinib after second‐line therapy, the most frequently observed adverse reactions were fatigue and gastrointestinal toxicity (fatigue 40% (grade 3: 6%, grade 4: 3%), anorexia 40% (grade 3: 6%, grade 4: 6%), and diarrhoea 34% (grade 3: 3%)) [9]. However, there are no reports on the safety of first‐line treatment with osimertinib in patients older than 75 years, but this is currently being investigated in a prospective study in Japan [10]. The incidence of gastrointestinal side effects is expected to increase during the first‐line treatment of elderly patients. We report a safe, long‐term response to the first‐line treatment with osimertinib for de novo *EGFR* T790M disease in elderly patients. If the efficacy of osimertinib for de novo *EGFR* T790M and its safety in the elderly are confirmed, it might be useful as a potential first‐line treatment.

### Disclosure Statement

Appropriate written informed consent was obtained for publication of this case report and accompanying images.

### Author Contribution Statement

Conceptualization: Toshiyuki Sumi. Investigation: Toshiyuki Sumi, Koki Kamada, Naoki Shijubou, and Yuichi Yamada. Writing—original draft: Toshiyuki Sumi. Writing—review and editing: Hisashi Nakata, Yuji Mori, and Hirofumi Chiba.
